# Quantitative SERS Detection of TBBPA in Electronic Plastic Based on Hydrophobic Cu-Ag Chips

**DOI:** 10.3390/bios12100881

**Published:** 2022-10-17

**Authors:** Pei Dai, Xianzhi Huang, Yaqian Cui, Lihua Zhu

**Affiliations:** 1 School of Chemistry and Chemical Engineering, Huazhong University of Science and Technology, Wuhan 430074, China; 2 Yellow Crane Tower Science and Technology Park (Group) Co., Ltd., Wuhan 430074, China

**Keywords:** tetrabromobisphenol A, surface-enhanced Raman scattering, hydrophobic interaction, bi-metal chips, synergistic coupling

## Abstract

Tetrabromobisphenol A (TBBPA) was one of the most widely used brominated flame retardants. However, it easily contaminates nature and harms the environment and human health during its production and use. Therefore, it is necessary to strictly control the content of TBBPA in electronics. Surface-enhanced Raman spectroscopy has the advantages of being fast and sensitive, but it is difficult to obtain the SERS spectra of TBBPA because the hydrophobic TBBPA molecule is difficult to approach with the hydrophilic surface of common noble metal SERS substrates. In the present work, a hydrophobic Cu-Ag chip was developed for the SERS detection of TBBPA. The integration of the hydrophobic interaction and the Ag-Br bonding promoted the adsorption of TBBPA on the Cu-Ag chip, allowing for SERS detection. It was observed that both the hydrophobicity and bimetallic composition of the Cu-Ag chip played important roles in the SERS detection of TBBPA. Under the optimized conditions, the low limit of detection of the established SERS method for TBBPA was 0.01 mg L^−1^, within a linear range of 0.1–10 mg L^−1^. Combined with ultrasonic-assisted extraction, the substrate could be used for the quantitative determination of TBBPA in electronic products. Compared with the HPLC-UV method used as a national standard, the relative error of the SERS method for quantifying the TBBPA content in a mouse cable and shell was ±3% and ±7.7%, respectively. According to the SERS results, the recovery of TBBPA in the spiked mouse shell was 95.6%.

## 1. Introduction

Tetrabromobisphenol A (TBBPA) is currently the most widely used brominated flame retardant (BFRs) worldwide [1,2], and is often used as an additive flame retardant in the electronic plastic resins ABS (Acrylonitrile-Butadiene-Styrene) and HIPS (High Impact Polystyrene). It accounts for more than 60% of the total output of BFRs, with an annual demand of nearly 200,000 tons [3]. With the continuous development of the manufacturing industry, the production and demand of flame retardants are increasing. However, TBBPA is highly polluting and persistent, and it interferes in the development of brain and bone, harms the endocrine and hormonal systems with long-time exposure [4]. Due to its volatilization during production and use, TBBPA can penetrate into the atmosphere, water and soil. With the circulation of matter and energy in the biosphere, it may be enriched and eventually endanger the life and health of various organisms [5]. According to Annex II of the RoHS Directive, the permitted limit of TBBPA as an additive flame retardant is 0.1 wt% in homogeneous materials, such as shell and packaging, and the reactive TBBPA in the circuit board may be exempted. Therefore, it is necessary to monitor and control the amount of TBBPA in these products.

Liquid chromatography [6,7,8,9] and gas chromatography [10,11] are the main detection methods of TBBPA at the present time. They can realize the accurate qualitative and quantitative detection of TBBPA, with the advantages of high sensitivity and reproducibility. However, the analysis methods based on chromatography still have problems, such as the high cost of equipment, the complex and tedious sample pretreatment and the long detection cycle. Moreover, derivatization of TBBPA is always needed for its detection by gas chromatography, which may lead to low recovery of TBBPA. Xie et al. [12] developed a GC-MS method for quantitative detection of TBBPA, and the recovery was only 79 ± 1%. Alternatively, the electrochemical method can provide a more effective strategy for TBBPA detection, having the advantages of low cost, high sensitivity, and being simple and convenient [13,14,15]. Zhao et al. [16] successfully detected TBBPA on a g-C_3_N_4_-modified glassy carbon electrode (GCE) with a limit of detection (LOD) of 5 nM. Wang [17] modified gold nanorob/polycysteine composite on a GCE and decreased the LOD to 3.2 nM. However, the electrochemical detection of TBBPA may have poor selectivity. This can be improved by combining it with molecularly imprinted technology [18] and electrochemical immunosensors [19].

Surface-enhanced Raman spectroscopy (SERS) can reflect the fingerprint information of target molecules [20,21]. It has been used in the rapid detection of organic molecules due to its high sensitivity, convenience and efficiency [22,23,24,25]. TBBPA has a large oil-water distribution coefficient (log K_ow_ = 4.5), showing strong hydrophobicity with a water solubility of only 4.15 mg L^−1^ (pH = 7). Therefore, TBBPA molecules are difficult to adsorb on the surface of noble metal hydrosols and other hydrophilic substrates to realize the SERS detection. Therefore, there are few reports on the SERS detection of TBBPA. Kadasala used magnetic Au nanoclusters modified with 4-dimethylaminopyridine (DMAP) as an enhanced substrate to realize the SERS detection of TBBPA with a LOD of 1 nM [26]. However, modification with the organic ligand DMAP introduced a large number of strong background peaks, seriously interfering with the discrimination of the characteristic peaks of TBBPA. We anticipated that the establishment of surface-clean hydrophobic SERS substrates can overcome the difficulties in the SERS detection of hydrophobic molecules.

In recent years, hydrophobic materials have attracted more and more attention. They have broad application prospects in metal corrosion prevention [27,28], self-cleaning [29,30], oil-water separation [31,32], surface-enhanced Raman spectroscopy [33,34] and other aspects. In general, the surface hydrophobicity of solid materials is mainly affected by two factors, the rough structure and chemical composition of the surface. Therefore, in addition to modifying low surface energy molecules on the material surface, hydrophobicity could also be realized by constructing a rough surface to form an air cushion which cannot be wet by water. There are many methods to prepare the hydrophobic materials, including the template method [35], the chemical etching method [36], the hydrothermal method [37], the electrodeposition method [38] and so on. Most of them have the problems of involving complex preparation processes and large instruments, and they cannot be applied in large scale production. To simplify the preparation process and develop an environmentally friendly preparation method, in the early work of our group, the superhydrophobic copper coating was prepared by a chemical reduction method and applied to the oil-water separation [39]. With the aid of hydrazine reduction without adding any modified agents, Cu NPs were deposited on the fabrics to form the superhydrophobic surface.

Therefore, aiming to achieve SERS detection of the hydrophobic TBBPA, we developed a hydrophobic Cu-Ag SERS chip to improve the SERS enhancement of substrate and its binding ability with TBBPA. Firstly, the hydrophobic copper-coated fabric with ordered micro-nano structure was prepared. Then, a hydrophobic Cu-Ag chip was constructed by introducing Ag onto the Cu surface through a replacement reaction. The synergistic coupling of Cu-Ag bimetal promised the SERS chips a good SERS enhancement. The surface of the Cu-Ag chips was hydrophobic and had abundant Ag. It made it so that TBBPA could approach the surface of the Cu-Ag chips based on the hydrophobic interaction, and finally bonded with the substrate through Ag-Br. Therefore, the SERS detection of TBBPA was realized with low interference and high sensitivity. Finally, the SERS method was combined with ultrasound-assisted extraction technology to achieve rapid quantitative determination of the TBBPA content in electronics.

## 2. Experimental Section

### 2.1. Reagents and Materials

AgNO_3_, N_2_H_4_·H_2_O (85%), citrate sodium, crystal violet (CV), tetrahydrofuran (THF), toluene, methanol and ethanol were purchased from Sinopharm Chemical Reagent Co., Ltd. (Shanghai, China). Cu(CH_3_COO)_2_·H_2_O was purchased from Shanghai Lingfeng Chemical Reagents Co., Ltd. (Shanghai, China). Sudan Ⅰ and Sudan Ⅲ were purchased from Macklin (Shanghai, China). TBBPA (98%) was purchased from Aladdin Chemistry Co., Ltd. (Shanghai, China). Nylon66 microporous filtration membranes were purchased from Tianjin Jinteng Experimental Equipment Co., Ltd. (Tianjin, China). Fabrics were made of ordinary pure cotton. All the chemicals were analytical grade and used without further purification. Deionized water was used throughout the experiments.

### 2.2. Preparation of Hydrophobic Cu-Ag Chips

The hydrophobic Cu-Ag chip was prepared with a modified chemical reduction method. Fabrics were firstly cut into pieces with a size of 2 × 2 cm, followed by washing with water and ethanol. After drying, it was immersed in a solution of Cu(CH_3_COO)_2_·H_2_O (20 mL, 10 g L^−1^) for several minutes. The Cu NPs were reduced and deposited on the fabric by adding hydrazine hydrate (800 μL, 80%) drop by drop until quiescence at room temperature for 24 h. Then, the Cu-coated fabric was taken out to wash with water and ethanol, and immersed in the ethanol solution of AgNO_3_ (0.1 M) for 5 min. After being washed again and vacuum dried at 60 °C for 1 h, the hydrophobic Cu-Ag chip was finally obtained for subsequent SERS detection.

The morphology of the obtained Cu-Ag chip was characterized by a GeminiSEM 300 scanning electron microscope (ZEISS, Oberkochen, Germany). Contact angles (CAs) were measured with an OCA20 contact angle instrument (Dataphysics, Filderstadt, Germany). X-ray diffraction (XRD) patterns were obtained with a SmartLab-SE diffractometer (Rigaku, Tokyo, Japan).

### 2.3. Preparation of Hydrophilic Ag Chips

To show the advantages of the above Cu-Ag chip in the SERS detection of hydrophobic targets, a hydrophilic Ag chip was prepared for comparison. Firstly, Ag NPs were prepared by a one-step reduction method with using sodium citrate as the reducing agent. The aqueous solution of AgNO_3_ (100 mL, 1.0 mmol L^−1^) was heated to boiling, and then sodium citrate (2.0 mL, 1 wt%) was quickly added to it. After the reaction proceeded for 30 min and the solution cooled to room temperature, the Ag nanoparticles were obtained. To prepare the Ag chip, the organic filter membrane (0.22 μm) was placed on the vacuum filtration device, then 5 mL Ag sol and 5 mL toluene were added into the container, on the filter membrane, to form the immiscible two-phase fluid. Then, 2 mL ethanol was injected into the interface of the two phases with a syringe, so that the Ag NPs self-assembled in the interface to form the Ag array. The vacuum pump was started to filter the solution, and the assembled Ag array was transferred to the filter membrane. After drying, the hydrophilic Ag chip was finally obtained for subsequent SERS detection.

### 2.4. Analysis of TBBPA

The SERS detection of TBBPA was achieved with the hydrophobic Cu-Ag chip. The Cu-Ag chip was cut into small pieces each with a size of 0.5 × 0.5 cm and immersed into the ethanol solution of target molecules for 10 min for further detection. SERS detection was performed with the ATR8100 microscopic Raman spectrometer (Optosky, Xiamen, China) with a 785 nm laser as the excitation source. The laser power was set to 50 mW, and the exposure time was 5 s. During the test, ethanol was dropped onto the SERS substrate to keep it wet. If not specified elsewhere, 5 different positions were randomly selected on each SERS chip for parallel tests, and the average spectrum was taken for further analysis.

To verify the accuracy of the SERS method, the content of TBBPA was also quantitatively detected by high-performance liquid chromatography with an HPLC-1260 (Agilent, Santa Clara, CA, USA). A ZORBAX SB-C18 reversed-phase column was used to separate the targets. The injection volume was 20 μL and the column temperature was 30 °C. The mobile phase was a mixture of methanol/water = 3/7 (*v*/*v*), with a flow rate of 1.0 mL min^−1^. The detection wavelength was set to 230 nm for the quantitative analysis.

### 2.5. Sample Pretreatment of the Electronics

To quantitatively detect the content of TBBPA in practical electronics, ultrasonic assisted extraction (UAE) was used to extract TBBPA from solid samples. Firstly, 0.1 g of electronic products were cut into small particles with a size less than 1 mm. UAE was performed with a KQ-600KDE ultrasonic cleaner (Shumei, Kunshan, China) at 240 W, using 5 mL tetrahydrofuran (THF) as the extractant. After 20 min of extraction, 15 mL methanol was added to the extraction solution to precipitate the plastic. Then, the organic filter was used to remove the precipitates, and the obtained filter liquor was retained for subsequent detection. The analytical methods for TBBPA detection were described in Section 2.3.

## 3. Results and Discussion

### 3.1. Characterization of the Cu-Ag Chip and Its SERS Detection of TBBPA

**Figure 1:**
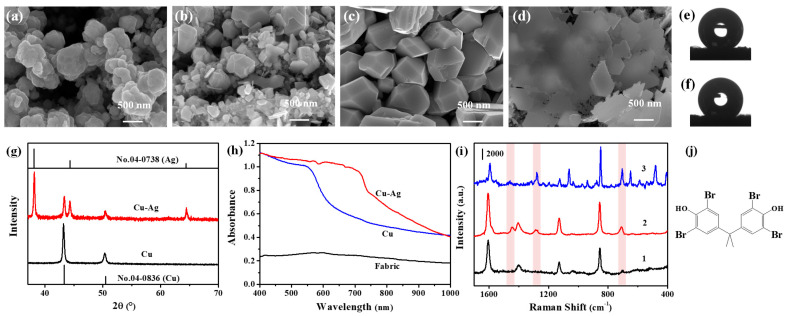
Characterization of Cu-Ag chips and their SERS detection of TBBPA. SEM images of the (**a**) Cu coating with 24 h deposition and (**b**–**d**) Cu-Ag coating with replacement times of (**b**) 3 min, (**c**) 5 min and (**d**) 10 min. (**e**,**f**) Contact angles of the coatings of (**e**) 24 h Cu and (**f**) 24 h Cu-5 min Ag on the fabric. (**g**) XRD patterns and (**h**) Vis-NIR diffuse reflectance spectra of the Cu and Cu-Ag chips. (**i**) Comparison between SERS spectra of (1) a blank background, (2) TBBPA solution (10 mg L^−1^) obtained from the hydrophobic Cu-Ag chips and (3) Raman spectrum of TBBPA powders. (**j**) The molecular structure of TBBPA.

In the present work, Cu NPs were firstly deposited on the fabrics through a chemical reduction method. By using hydrazine hydrate as the reducing agent, Cu NPs were deposited uniformly and closely on the fabric. Figure 1a shows the SEM image of the surface morphology of the Cu coating with a deposition time of 24 h (this Cu coating was referred to as the 24 h Cu chip). It was observed that the spherical Cu NPs with diameters of 300–500 nm were packed tightly together. The structure of the Cu coating made the fabric hydrophobic, and the contact angle of the water droplet on its surface was about 149° (Figure 1e). As shown in Figure 1g, the XRD pattern of the Cu coating showed two peaks at 43.3° and 50.5°, which matched well with the diffraction peaks of (1 1 1) and (2 0 0) of Cu (JCPDS 04-0836), indicating that the state was that of metallic copper. Therefore, Ag could be deposited on the surface of the Cu metal through the replacement reaction.

Compared with the Cu chip, the XRD pattern of the Cu-Ag chip (Figure 1g) showed three new peaks appearing at 38.1°, 44.3° and 64.4°, corresponding to the (1 1 1), (2 0 0), and (2 2 0) crystal planes of silver (JCPDS 04-0783), respectively. Along with the prolonging of replacement reaction time, the size of the Ag nanoparticles on the 24 h Cu coating increased. As shown in Figure 1b–d, with increases in the replacement time of Ag from 3 min to 10 min, the small-size Ag NPs (Figure 1b) gradually became too large to completely coat the Cu NPs (Figure 1c), and finally formed the Ag sheet structure at the micrometer scale (Figure 1d). For the 24 h Cu-5 min Ag chip, Ag particles completely covered the underlayer Cu NPs and presented a multilateral shape of 500~800 nm. Although its surface roughness decreased, the tight packing of Cu-Ag NPs made the Cu-Ag chip still able to retain good hydrophobic properties, with a contact angle of 140.1°. Figure 1h shows the Vis-NIR diffuse reflectance spectra of the above Cu and Cu-Ag chips. The surface plasmon resonance (SPR) peak of the Cu chip appeared at 557 nm, and the Cu-Ag chip showed stronger SPR absorption with a red shift to 716 nm.

The SERS response of the TBBPA solution (10 mg L^−1^) was detected with the 24 h Cu-5 min Ag chip, as shown in Figure 1i. The background interference of the Cu-Ag chip was little, and the four peaks at 1607 cm^−1^, 1405 cm^−1^, 1129 cm^−1^ and 856 cm^−1^ might be attributed to some organic compounds of the fabric in the chip. The introduction of TBBPA solution introduced three new peaks at 1446 cm^−1^, 1283 cm^−1^ and 710 cm^−1^ in the SERS spectrum, consistent with the normal Raman peak of TBBPA solid. This indicated that the Cu-Ag chip without modification could directly adsorb TBBPA molecules and realize their SERS detection.

### 3.2. Effects of the Preparation Conditions of the Cu-Ag Chip on Its Performance

**Figure 2:**
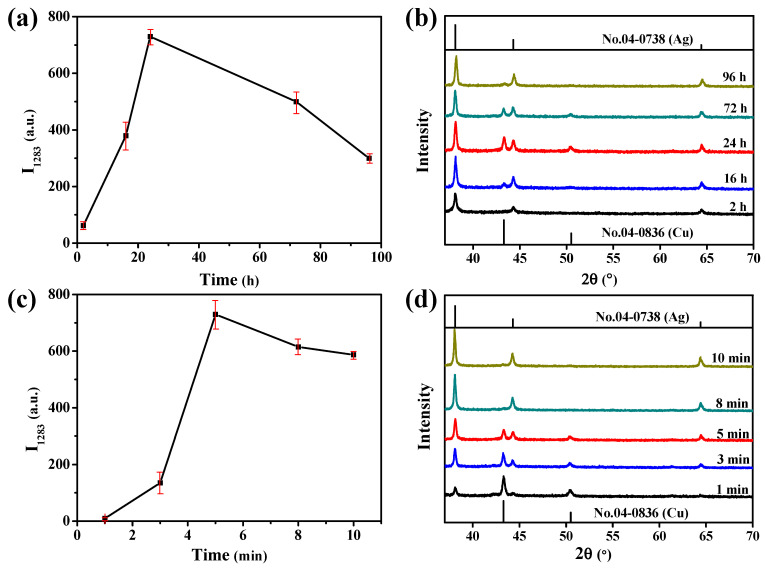
(**a**,**b**) Influence of the Cu deposition time on (**a**) the peak intensity of TBBPA at 1283 cm^−1^ and (**b**) the XRD pattern of the Cu-Ag chips. The replacement time of Ag was set as 5 min. (**c**,**d**) Influence of the Ag replacement time on (**c**) the peak intensity of TBBPA at 1283 cm^−1^ and (**d**) the XRD pattern of the Ag-Cu chips. The deposition time of Cu was set as 24 h.

The introduction of Ag in the Cu-Ag SERS chip increases not only the electromagnetic enhancement of the Cu-Ag chip but also the Ag-Br interaction between the SERS substrate and TBBPA. The deposition amounts of Cu and Ag were controlled by changing the deposition time of Cu and the replacement time of Ag, respectively. As shown in Figure 2a, when the replacement time for depositing Ag was fixed at 5 min, the SERS intensity of TBBPA on the Cu-Ag chip gradually increased with increases in the Cu deposition time. When the Cu deposition time was as short as two hours, all the deposited copper would be completely dissolved by the replacement reaction during the Ag deposition, so that the diffraction peaks of Cu were hardly observed on the XRD pattern of the 2 h Cu-5 min Ag chip (Figure 2b). When the Cu deposition time was extended to 24 h, the SERS intensity of TBBPA was the strongest. With further extensions to the Cu deposition time, the SERS intensity of TBBPA decreased. This may be due to the decrease in the relative content of Ag in the SERS substrate, which led to the weakening of both the SERS enhancement and the interaction of Cu-Ag chip with TBBPA.

Then, the influence of Ag replacement time on the SERS intensity was investigated by fixing the Cu deposition time at 24 h. As shown in Figure 2c, by prolonging the Ag replacement time, the peak intensity of TBBPA at 1283 cm^−1^ first increased and then decreased, and reached the maximum value at 5 min. Figure 2d shows the XRD pattern of Cu-Ag chips with different replacement times. As the replacement time increased from 1 min to 5 min, the Ag content on the SERS substrate increased continuously, and the characteristic peaks of metallic Cu could still be observed. This proved that the obtained SERS substrate was composed of Cu and Ag composite materials. When the replacement time was further extended to more than 5 min, Cu NPs were completely dissolved, and the SERS substrate became a monometallic Ag material. Therefore, the surface state had basically remained unchanged (Figure 2d). The contact angles of the Cu-Ag chips with replacement times of 8 min and 10 min were 143.2° and 140.0° respectively, which seemed to be nearly consistent with those at 5 min.

Among these SERS substrates prepared under different conditions, the 24 h Cu-5 min Ag chip had the strongest SERS enhancement for TBBPA. This could be explained by the above SEM and diffuse reflectance spectroscopy characterizations (Figure 1). Ag of the 24 h Cu-5 min Ag chip formed a polygonized structure with corner angle on the surface of the Cu, its tip provided great SERS performance. The core-shell composite structure also provided the synergistic coupling of Cu and Ag, resulting in the red shift of its SPR peak, which matched well with the 785 nm excitation laser. In addition, the Cu-Ag chip still maintained hydrophobicity under these preparation conditions, and the exposure surface of Ag increased. Both of them enhanced the adsorption capacity of the 24 h Cu-5 min Ag chip to TBBPA. Based on the above discussions, the increase in Ag content improved the Ag-Br interaction and the electromagnetic enhancement of the Cu-Ag substrate for TBBPA, so as to realize the SERS detection of TBBPA. The existence of a small amount of Cu could further enhance the SERS intensity of TBBPA on this Cu-Ag chip. Therefore, in the present work, the Cu deposition time and Ag replacement time were selected to be 24 h and 5 min, respectively.

### 3.3. Effect of the Hydrophobicity of the Cu-Ag Chip on the Detection of Hydrophobic Targets

**Figure 3:**
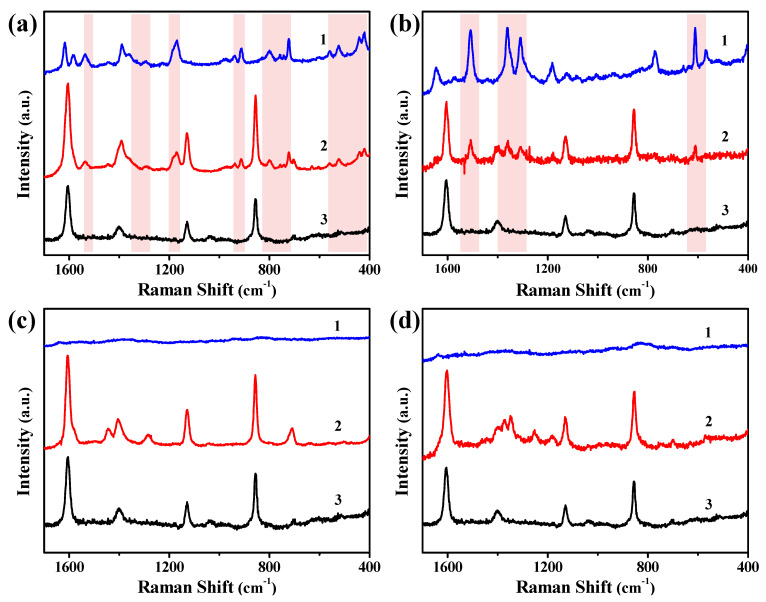
SERS spectra of the hydrophilic targets of (**a**) CV, (**b**) R6G, (**c**) TBBPA, and (**d**) Sudan Ⅰ obtained from the hydrophilic Ag chips (1) and the hydrophobic Cu-Ag chips (2), with the blank background of hydrophobic Cu-Ag chips (3) for comparison.

In order to evaluate the influence of the hydrophobicity of the Cu-Ag chip on the SERS detection of TBBPA, a completely hydrophilic Ag chip with a contact angle of 0° was prepared by interface assembly and transfer for comparison with the above hydrophobic Cu-Ag chip. Figure 3a,b gives the SERS responses of two hydrophilic targets, crystal violet (CV) and rhodamine 6G (R6G). Except for the four peaks of the blank background (1607 cm^−1^, 1405 cm^−1^, 1129 cm^−1^ and 856 cm^−1^), there were abundant characteristic peaks of target molecules in their SERS spectra obtained from the hydrophobic Cu-Ag chips. The SERS peaks marked in the pink area were the fingerprint peaks of CV and R6G. It could be found that the fingerprint peaks obtained from the Cu-Ag chips were consistent with the results obtained from the hydrophilic Ag chips, and the signal intensity was slightly lower than that of the hydrophilic substrate. This indicated that the hydrophobic Cu-Ag chip was applicable to the hydrophilic targets with good dispersion in solvent, and its SERS detection of target molecules was universal. The enhancement factor (EF) of CV on the Cu-Ag chips was 4.8 × 10^6^. The hydrophilic Ag chip had a good affinity for the hydrophilic target; its SERS performance was similar to that of the hydrophobic Cu-Ag chip.

In contrast, when the two substrates were applied for the SERS detection of the hydrophobic targets (as shown in Figure 3c,d), TBBPA and Sudan I dye, there was no characteristic peak in their SERS spectra obtained from the hydrophilic Ag chip. However, all their characteristic peaks could be observed in their SERS spectra obtained from the hydrophobic Cu-Ag chips. The EF of TBBPA on the Cu-Ag chips was 5.3 × 10^7^. These results showed that the hydrophobicity of the Cu-Ag chips could improve their adsorption of hydrophobic targets, so as to realize the SERS detection of the hydrophobic targets with weak binding ability. This was probably because the hydrophobic targets existed as polymolecular aggregates in the ethanol solution, which had weak binding force with the noble metal. Therefore, they were hardly adsorbed on the hydrophilic Ag substrate. These aggregate droplets could approach the surface of the Cu-Ag chip based on the hydrophobic interaction, and then adsorbed on the substrate through the Ag-Br bond; the SERS detection of TBBPA was finally realized on this hydrophobic Cu-Ag chip.

### 3.4. Quantitative SERS Detection of TBBPA in Electronic Products

**Figure 4:**
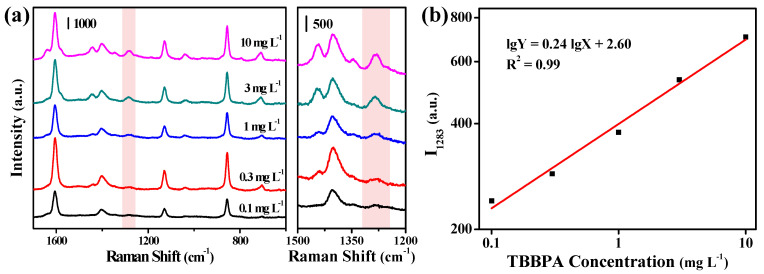
(**a**) SERS spectra of TBBPA in the different concentrations obtained from the hydrophobic Ag-Cu chips. (**b**) Standard curve for quantitative SERS detection of TBBPA from the hydrophobic Ag-Cu chips.

The Cu-Ag chip was used for quantitative detection of TBBPA, and the SERS spectra of TBBPA at various concentrations are shown in Figure 4a. It could be observed that the SERS intensity of the characteristic peaks of TBBPA increased gradually with the increase in its concentration. By taking the characteristic peak of TBBPA at 1283 cm^−1^ as the representative, the SERS intensity was plotted against the concentration of TBBPA (Figure 4b). It was found that there was a good linear relationship between the SERS intensity and TBBPA concentration on a logarithmic scale within the range of 0.1 to 10 mg L^−1^. The wide linear range and low LOD (0.01 mg L^−1^) indicated that the use of Cu-Ag chips provided a good quantitative analysis method for TBBPA.

Our previous work reported a method that could completely extract brominated flame retardants in electronics through ultrasonic assisted extraction with THF as the extraction agent [40]. By combining that extraction method and the presently developed SERS method based on the hydrophobic Ag-Cu chip, we realized the rapid quantitative detection of TBBPA in electronics. In order to verify the accuracy of the SERS method, we also used the HPLC-UV method as the national standard detection method of TBBPA to quantitatively detect the TBBPA content in the same mouse samples. As shown in Table 1, according to the SERS method, the content of TBBPA in the cable was 1980.1 mg kg^−1^, showing a relative error of −3.0% in comparison with the detection result (2040.3 mg kg^−1^) of the HPLC-UV method. As for the mouse shell sample, the detected content of TBBPA by the SERS and HPLC-UV methods was 316.9 mg kg^−1^ and 343.5 mg kg^−1^, respectively, with a relative error of −7.7%. All of these detected TBBPA contents were much lower than the permitted limit of TBBPA (0.1 wt%, or 1 g kg^−1^) in electronics [41].

The feasibility and accuracy of the UAE extraction-SERS detection method for quantitative detection of TBBPA in actual electronics were also verified through the spiked samples. Firstly, the spiked shell (spiked amount of 1500 mg kg^−1^) was prepared by soaking the mouse shell in the standard solution of TBBPA followed by drying the solvent. After the same extraction operation, the TBBPA content of the spiked shell was detected to be 1750.9 mg kg^−1^ and 1899.0 mg kg^−1^ by the SERS and HPLC-UV methods, respectively. Compared to the original content of TBBPA in shell, the spiked amount was calculated as 1434.0 mg kg^−1^ and 1555.5 mg kg^−1^, with recoveries of 95.6% and 103.7%, respectively. This proved that the ultrasonic-assisted extraction method had high extraction efficiency for the spiked TBBPA in the mouse shell, and the detection result of SERS method was accurate to use for the quantitative detection of TBBPA in electronic products.

## 4. Conclusions

A hydrophobic Cu-Ag chip was prepared for the SERS detection of TBBPA as a representative of hydrophobic substances. The hydrophobicity of the Cu-Ag chip enhanced the affinity of the substrate for the hydrophobic targets (such as TBBPA), which let TBBPA close to the surface of SERS substrate and then bonded with it through Ag-Br interactions. In addition, the synergistic coupling between the Cu and Ag bimetals improved the SERS enhancement of the substrate. These made it possible for the SERS detection of TBBPA on the Cu-Ag chip. By combining it with-ultrasonic assisted extraction (UAE), the quantitative detection of TBBPA in the electronics was realized. After UAE, the TBBPA content in electronics could be completely extracted and accurately determined, and the LOD of TBBPA was 2.0 mg kg^−1^ (0.01 mg L^−1^ for the TBBPA solution). Compared with the HPLC-UV, the SERS method was quick, convenient and sensitive; the quantitative detection results could be used to distinguish if the TBBPA content in electronics was appropriate.

## Figures and Tables

**Table 1:** TBBPA contents in the mouse and its spiked sample determined by SERS and HPLC-UV methods.

Sample (mg kg^−1^)	SERS	HPLC-UV
Cable	1980.1	2040.3
Shell	316.9	343.5
Spiked shell	1750.9	1899.0

## Data Availability

Not applicable.
